# Modeling the Effects of Harvest Alternatives on Mitigating Oak Decline in a Central Hardwood Forest Landscape

**DOI:** 10.1371/journal.pone.0066713

**Published:** 2013-06-18

**Authors:** Wen J. Wang, Hong S. He, Martin A. Spetich, Stephen R. Shifley, Frank R. Thompson III, Jacob S. Fraser

**Affiliations:** 1 School of Natural Resources, University of Missouri, Columbia, Missouri, United States of America; 2 Arkansas Forestry Sciences Laboratory, USDA Forest Service, Southern Research Station, Hot Spring, Arkansas, United States of America; 3 USDA Forest Service, Northern Research Station, Columbia, Missouri, United States of America; DOE Pacific Northwest National Laboratory, United States of America

## Abstract

Oak decline is a process induced by complex interactions of predisposing factors, inciting factors, and contributing factors operating at tree, stand, and landscape scales. It has greatly altered species composition and stand structure in affected areas. Thinning, clearcutting, and group selection are widely adopted harvest alternatives for reducing forest vulnerability to oak decline by removing susceptible species and declining trees. However, the long-term, landscape-scale effects of these different harvest alternatives are not well studied because of the limited availability of experimental data. In this study, we applied a forest landscape model in combination with field studies to evaluate the effects of the three harvest alternatives on mitigating oak decline in a Central Hardwood Forest landscape. Results showed that the potential oak decline in high risk sites decreased strongly in the next five decades irrespective of harvest alternatives. This is because oak decline is a natural process and forest succession (e.g., high tree mortality resulting from intense competition) would eventually lead to the decrease in oak decline in this area. However, forest harvesting did play a role in mitigating oak decline and the effectiveness varied among the three harvest alternatives. The group selection and clearcutting alternatives were most effective in mitigating oak decline in the short and medium terms, respectively. The long-term effects of the three harvest alternatives on mitigating oak decline became less discernible as the role of succession increased. The thinning alternative had the highest biomass retention over time, followed by the group selection and clearcutting alternatives. The group selection alternative that balanced treatment effects and retaining biomass was the most viable alternative for managing oak decline. Insights from this study may be useful in developing effective and informed forest harvesting plans for managing oak decline.

## Introduction

For more than a century, oak decline and associated mortality have occurred in the oak forests of the eastern United States. Since the late 1990s, oak decline has become a prominent problem throughout the Ozark Highlands of Missouri, Arkansas, and Oklahoma [1].The most recent oak decline events occurred from 1999 to 2005 and severely affected approximately 12,000 ha in the Ozark National Forest of Arkansas alone [2]. Oak decline related to pathology typically begins with foliage wilting and browning followed by progressive branch dieback and tree mortality. Oak decline in the Eastern U.S is caused by complex interactions of predisposing factors, inciting factors, and contributing factors [3]–[5]. Stands are predisposed to oak decline by high tree density, species composition, advanced tree age, and shallow and rocky soils. Inciting factors including severe drought or insect defoliation can stress oaks into decline. Contributing factors, such as insects and pathogens also can impact trees already under stress and thus further increase rates of mortality.

Red oak group (*Quercus* section *Lobatae*) species including black oak (*Q. velutina* Lam.), northern red oak (*Q. rubra* L.), and scarlet oak (*Q. coccinea* Muenchh.) are more susceptible to oak decline than white oak group (*Quercus* section *Quercus*) species [5]–[8]. This is especially true for red oaks (referring to red oak group species) that are physiologically mature and growing on shallow rocky soils, ridges, or south and west-facing slopes [9]. During early 1900s, these low quality sites often favored the establishment of red oaks after extensive timber harvesting [10]. Because older red oaks growing on these draughty, low quality sites become especially stressed when competing for limited water and nutrients and old-age trees have less capacity to counteract stress and resume growth, oak decline therefore is more prominent in stands dominated by older red oaks on low quality sites [11].

Oak decline in affected areas has dramatically altered species composition and stand structure, degraded timber value, reduced wildlife habitat quality, and increased fuel load. In one of the most severely impacted forests of northern Arkansas, total overstory basal area and density were reduced from 24 to 13 m^2^/ha and from 385 to 220 trees/ha, respectively; basal area and density of overstory red oaks were reduced from 12 to 3 m^2^/ha, and from 148 to 27 trees/ha, respectively [12]. Heitzman et al. [13] also found that oak decline resulted in a shift in species importance from what once were red oak-dominated stands to more mixed stands of white oak (*Q. alba* L.), red oaks, hickory (*Carya spp*.), blackgum (*Nyssa syvatica* Marsh.), and red maple (*Acer rubrum* L.).

Forest harvesting has been widely advocated to reduce or prevent exposure to predisposing factors in oak decline by removing susceptible species and declining trees [14]–[16]. Prior research has examined the effects of forest harvesting on managing oak decline at stand scales over relatively short-time frames (e.g. less than 20 years). Three widely used harvest alternatives for reducing vulnerability to oak decline are: clearcutting, group selection, and thinning [14], [16]. Burrill et al. [17] showed that thinning conducted in even-aged stands before reaching rotation age was useful in preventing future oak decline by increasing stand vigor and controlling species composition. Fan et al. [18] showed that oak decline typically occurred in stands at the understory reinitiation stage, thus they recommended that marking trees for harvesting should focus on those with a high probability of mortality. Shifley et al. [5] showed that tree crown class, diameter, and basal area of larger non-red-oaks explained most of the variability in oak mortality. They thus recommended that large co-dominant red oaks should be given highest priority for harvesting because of their high economic value and susceptibility to mortality associated with oak decline. Clearcutting may be an option for managing oak decline in stands that are largely comprised of red oaks. Group selection provides the option of removing patches of vulnerable trees or scattered declining trees [16], [19].

Such stand-scale studies provide a scientific basis for applying stand-scale silvicultural treatments to mitigate oak decline, but are insufficient for addressing long-term cumulative management effects at broad spatial and temporal scales. Oak decline is a spatially contiguous landscape process driven by a variety of processes operating from stand to landscape scales [20]. At stand scales, ecological succession and related changes in species composition and stand structure can affect the current and future dynamics of oak decline. At landscape scales, the shifting mosaic of species composition and age cohorts caused by fire, forest harvesting, and environmental heterogeneity (e.g. slope and aspect) can also affect oak decline dynamics. Spetich and He [20] argued that the spatio-temporal patterns of oak decline provided the basis for where, when, how often, and what management alternatives should be used. Moreover, because tree age and species composition considered as predisposing factors in oak decline are temporally dynamic, this requires addressing oak decline over the long term [16]. Although much attention has been paid to maintaining long-term forest productivity and health at large spatial scales, comparatively little attention has been paid to evaluating the effects of forest harvesting on oak decline at landscape scales.

The objectives of this study were to (1) assess potential, landscape-scale oak decline risk using stand-scale experimental studies, (2) delineate the distribution of current and future potential oak decline risk sites, and (3) evaluate the effects of harvest alternatives including clearcutting, thinning, and group selection on mitigating oak decline at the landscape scale. The overall hypothesis was that harvest alternatives combing stand-scale silvicultural treatments with landscape-scale considerations (e.g., site selection and treatment allocation) should be effective in reducing the potential oak decline. Specifically, we hypothesized that when harvest intensity (total area harvested each year) was fixed because of limited fiscal and human resources, prioritizing high stand density and older red oak stands for harvesting should be effective in mitigating oak decline. To accomplish these objectives, we applied a spatially explicit forest landscape model (FLM), LANDIS PRO to assessing spatial and temporal variations in species composition and stand structure, and in the potential oak decline risk sites under the three different harvest alternatives.

## Methods

### Study area

**Figure 1:**
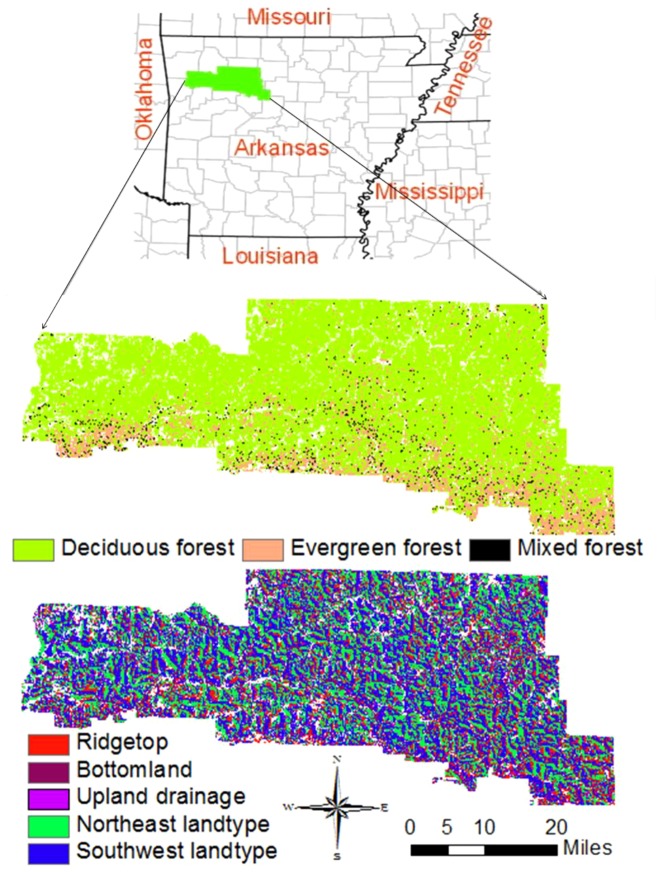
The study area is located in the Boston Mountains of Arkansas. These are predominantly hardwood forests dominated by oaks. This area is highly topographically dissected containing a variety of landtypes.

The study area is located in the Boston Mountains of Arkansas, which covers 427,660 ha (Figure 1). The area is the southernmost part of the Ozark Highlands of Central Hardwood Forest Region [21]. Most of the area belongs to Ozark-St. Francis National Forest. This mountainous area is deeply dissected and rugged, with elevations ranging from 275 m to 762 m. Average annual temperature and precipitation range from 14 to 17°C and from 1150 to 1325 mm, respectively, with the most of the rainfall occurring in spring and fall. Soils in the region are mostly Udults. Most of this area is covered by hardwood forests composed various mixtures of white oak, post oak (*Q. stellata* Wangenh.), chinkapin oak (*Q. muehlenbergii* Engelm.), black oak, northern red oak, blackjack oak (*Q. marilandica* Muenchh.), southern red oak (*Q. falcate* Michx.), and scarlet oak. Pignut hickory (*C. glabra* Sweet.) and black hickory (*C. texana* Buckl.) are commonly associated with oaks. Shortleaf pine (*Pinus echinata* Mill) is abundant in the southern part of the study area.

Species composition and distribution in this area have been significantly altered since European settlement [10], [22]–[23]. Red oaks are now more abundant than they were during the pre-settlement era, most of which regenerated following the extensive timber harvesting in the early 1900s. These red oak-dominated stands are now at or near maturity ranging from 70 to 100 years. Stand densities are relatively high because of nearly a century of fire suppression. The high stand density and mature age in combination with a drought from 1998 to 2000 and repeated insect defoliation have made oak forests in this area more conductive to oak decline [24]. Severe oak decline has dramatically affected oak forests in this region. Spetich [25] stated that during just one year, the basal area of dead trees increased from 1.8 to 4.4 m^2^/ha and the basal area of living trees decreased by 2.9 m^2^/ha from 2000 to 2001. Therefore, there is a great need to develop effective management alternatives for reducing susceptibility of forests to oak decline and for improving forest health throughout this region. There was no specific permission for our current study area -Boston Mountains, Arkansas, because our study research was funded by USDA Forest Service, Southern Research Station to study oak decline in this area. In addition, our field studies did not involve endangered or protected species.

### Forest landscape model

We used the LANDIS PRO FLM [26] to simulate forest succession and harvest alternatives to evaluate the potential oak decline risk. LANDIS PRO is a raster-based FLM; within each raster cell, the model records number of trees by species age cohort and size (e.g., DBH) for each species age cohort is derived from empirical age-DBH relationships. LANDIS PRO can estimate density, basal area, and biomass by species for all raster cells on a modeled landscape. It is highly compatible with forest inventory data, thus extensive forest inventory data can be directly utilized to initialize and constrain model parameters. LANDIS PRO simulates forest change by incorporating species-, stand-, and landscape-scale processes. Species-scale processes include tree growth, establishment, and mortality and are simulated using species’ vital attributes and empirical age-DBH relationships. Stand-scale processes include density- and size-related resources competition that regulates self-thinning and seedling establishment. The competition intensity is quantified by growing space occupied (GSO) estimated by the percentage of the total minimum growing space required by all trees in a raster cell. The minimum growing space is derived from the Reineke stand density index (SDI) [27] and maximum SDI using tree density and size information.

Stand development patterns are governed by GSO and are simulated to follow the well documented stages: stand initiation, stem exclusion, understory reinitiation, and old-growth stage [28]. Seedlings can only become established before stands reach fully occupied in the stand initiation stage depending on species’ shade tolerance and species’ establishment probability (SEP). Once stands exceed maximum growing space occupied (MGSO), stands reach the stem exclusion stage. Meanwhile, self-thinning is initiated and continues to the following the understory reinitiaion and old-growth stages [28]. LANDIS PRO implements self-thinning using Yoda’s self-thinning line [29], where the tree mortality is characterized by a decrease in the number of trees with increasing average tree size in the stand and follows the -3/2 rule. Trees that are small, shade intolerant, or approaching their longevity can be outcompeted first via self-thinning [30]. As the mean size of trees in the stand increases, larger canopy gaps are created by the death of trees. During the understory reinitiaion stage, these gaps are refilled by establishment of new seedlings. Continued tree growth and mortality in the absence of exogenous disturbance move the stand into the old-growth stage, where old trees die as they reach their longevity, creating large canopy gaps that promote tree regeneration and move the stand into uneven-aged condition.

Landscape-scale processes include management (forest harvesting, prescribed fire), natural disturbance, and seed dispersal. To account for heterogeneity across the landscape, the landscape is stratified into relatively homogeneous units called landtypes reflecting variation in the physical environment. Within a given landtype, similarities in SEP and resource availability (MGSO) are assumed. Since SEP and MGSO vary spatially and temporally, they are capable of reflecting landscape heterogeneity in space and time.

Forest harvesting is simulated using LANDIS PRO harvest module [31]. In the harvest module, forest harvesting is simulated using a management area map and a stand map. Each management area in the management area map provides a boundary where certain specified harvest event can occur. Stands in the stand map are delineated to reflect the real forest stands on the ground. Each stand encompasses a group of raster cells and is a smaller contiguous unit within each management area. The total amount of area for harvesting is user-specified as percentage of each management area. Harvest events can occur at any time step and have the option of reoccurring at a user-specified time interval. When a harvest event is triggered within a management area, specific stands in this management area are prioritized for harvesting based on user-specified ranking criteria (e.g., basal area or stocking) until the amount of harvest set by users is satisfied. Three types of harvest event can be simulated in the harvest module: thinning, clearcutting, and group selection. Thinning intensity is determined by a user-specified residual basal area or stocking for the selected stands; stands can be thinned staring from largest tree or smallest tree. Thinning events that have a residual basal area or stocking value of zero are simulated as clearcutting. Group selection is simulated to create canopy openings in the stand map. The mean opening size for group selection is user-specified as number of raster cell within each stand. Once group selection is triggered within a stand, all trees in the raster cells determined by opening size within the stand are harvested.

### Using forest inventory data to initialize the landscape predisposed to oak decline

Eleven of the most common tree species in this study area were grouped into six species functional groups, which accounted for 90% of total basal area: white oaks (white oak, post oak, and chinkapin oak), red oaks (northern red oak and southern red oak), black oak, hickories, pines (shortleaf pine and loblolly pine (*P. taeda* L.)), and maples (red maple and sugar maple (*A. saccharum* Marsh)). The species’ vital attributes (Table 1), landtype map, and species’ establishment probability by landtype were compiled based on existing data sets for the Boston Mountains [20] and Silvics of North America [32]. All the input maps were gridded to a 90 m cell size.

**Table 1:** Calibrated species’ vital attributes used in the application of LANDIS PRO in the study area compiled based on existing data sets for the Boston Mountains [20] and Silvics of North America.

Species group name	Longevity (years)	Mean maturity (years)	Shade tolerance (class)	Fire tolerance (class)	Maximum seeding distance(m)	Vegetative reproduction probability	Minimum sprouting age(years)	Maximum sprouting age (years)	Maximum DBH (cm)	Maximum SDI (trees/ha)	Number of potential germination seeds
Pine	200	20	3	4	200	0.5	1	47	60	990	50
Black oak	120	20	3	3	200	0.4	10	70	60	570	90
Red oak	150	20	3	3	200	0.4	10	70	60	570	90
White oak	300	20	4	4	200	0.5	10	50	65	570	90
Hickory	250	20	3	3	200	0.5	10	70	60	570	30
Maple	200	20	5	1	200	0.3	10	70	60	570	90

We created the initial forest species composition map (1978) for the study area containing number of trees by species age cohort in each cell from 1978 U.S. Forest Service Inventory and Analysis (FIA) data, which contained 2042 plots and 16,000 individual tree records. To verify how well the initialized forest composition map represented the historical forest conditions, we iteratively adjusted species growth rates (the curve of DBH increment with age) until the initialized basal area by species group matched with the summarized FIA data of 1978 at both the landscape and landtype scales (e.g., northeast and southwest landtypes). Ideally such comparisons are conducted using independent data. However, independent spatial and temporal data are not often available at landscape scales. Thus, in our study, we used a data-splitting approach to avoid using the same data for initialization and comparison. Specifically, we used half of 1978 FIA data for initializing forest landscape and reserved the other half of the data to compare with the initialized landscape to ensure our initialized landscape matched with the historical forest conditions of 1978.

The initialization was conducted using the Landscape Builder software, which was developed specifically for LANDIS PRO [33]. Landscape Builder first converted the tree-level inventory data for species and diameter within each FIA plot to number of trees per hectare by species age cohorts using published DBH-age equations (e.g., Loewenstein et al. [34]). These data were then scaled for individual cells based on the cell size and FIA tree expansion factor [35]. Third, Landscape Builder stochastically selected and assigned a representative FIA plot to each cell to represent species composition for the initial forest conditions. Each FIA plot assigned to represent a particular cell was screened and stratified to draw only from the pool of FIA plots that matched the cell in FIA unit, national forest type, and national forest size class.

The initialized landscape for 1978 was then used as the starting point to simulate forest succession and dynamics without disturbance to year 2008 (30 simulation years). We further calibrated number of potential germination seeds, a model parameter influencing density and basal area until the predicted density and basal area by species group at 2008 matched with the observed changes in the FIA inventory for the same time period at landscape and landtype scales. Some degree of dependence may exist given that data were recorded in two time periods only 30 years apart. However, these data did provide a rare record of observed changes over three decades allowing for calibrating model parameters. After calibrating the model, we created the initial landscape for 2008 using 2008 FIA data. We then simulated forest landscape change for from 2008 to 2108 (100 years) using the calibrated model parameters.

### Oak decline risk rating

Because forest harvesting concentrated on reducing exposure to predisposing factors, predisposing factors should be considered when evaluating the effects of the harvest alternatives on mitigating oak decline. We synthesized the prior stand-scale experimental research to rate potential risks for oak decline based on the predicted basal area of red oaks and site quality [36]–[37]. Site quality was derived from landtype (e.g., south and ridgetop were classified as low quality sites). Since a south or ridgetop landtype may result in high oak decline risk for the entire facet, landtype served as a spatial control that accounted for the influence of spatial patterns on oak decline risk rating. Together, the predicted basal area of red oaks and site quality jointly quantified potential risk accounting for both biotic and abiotic causes [8], [16]:

1. High risk: stands on low quality sites including ridgetops and southwest-facing slopes with >6.9 m^2^/ha of red oak basal area; or high quality sites including floodplain and northeast-facing slope with >13.8 m^2^/ha of red oak basal area;
2. Moderate risk: stands on sites including ridgetops and southwest facing slopes with 2.3- to 6.9 m^2^/ha of red oak basal area; or high quality sites (including floodplain and northeast-facing slopes) with 2.3- to 13.8 m^2^/ha of red oak basal area;
3. Low risk: stands with less than 2.3 m^2^/ha of red oak basal area.

Sites were mapped and summarized by oak decline risk category as a percentage of the total landscape (number of pixels divided by the total number of pixels). Then, the three response variables quantifying oak decline were expressed as the percentage of area in high risk, moderate risk, and low risk sites. Since management for mitigating oak decline rarely focuses on low risk sites, we chose the high- and moderate-risk sites for oak decline at simulation periods of 20, 50, 100 to represent the short-, medium, and long-term responses.

### Experimental design

#### Harvest alternatives

Key factors of forest harvesting at landscape scales included the selection of: 1) treatment sites and their spatial allocation, 2) tree species and their ages for harvesting, and 3) treatment types (clearcutting, thinning, and group selection). Earlier studies suggested that management for oak decline should focus on low quality sites [13]. High density stands were given highest priority for harvesting because of intensive competition for resources. Red oaks of older ages were given highest priority for harvesting because of their susceptibility to decline [18]. In our study, we applied all three treatment types to low quality sites, but thinning was applied only to high quality sites (e.g. floodplains and northeast-facing slopes). In total, there were four harvest alternatives with a single factor (treatment type): (1) clearcutting, (2) group selection, (3) thinning, and (4) no-harvest. For each harvest alternative, we simulated 100 years of forest change for the entire study area using a five-year time step and each alternative was replicated five times. The harvest parameters including harvest rotation, harvest intensity (percent area harvested each year), and residual basal area were derived from the current management plan of Ozark- St.Francis National Forests (Table 2) [38]. Eighteen management areas were parameterized in the study area based on management goals and site quality.

**Table 2:** Harvest parameters for the thinning, clearcutting, and group selection alternatives in the applications of LANDIS PRO harvest module in the study area.

Thinning parameters	
#Management Area ID#	8 (e.g.)
#Ranking algorithm for stand selection:1is random stand selection, 6 is highest average basal area#	6
#Entry year#	5
#Reentry year#	5
#Minimum stand harvest basal area (m^2^)#	18.36
#remove largest tree first#	1
#Proportion of management area to harvest#	0.03
#Target stand basal area (m^2^)#	18.36
#Species priority ranking for harvest#	
#Pine#	6
#Black oak#	3
#Red oak#	4
#White oak#	5
#Hickory#	1
#Maple#	2

Clearcutting parameters	
#Management Area ID#	6 (e.g.)
#Ranking algorithm#	6
#Entry year#	5
#Reentry year#	5
#Minimum stand harvest basal area(m^2^)#	16
#remove largest tree first#	1
#Proportion of management area to harvested#	0.05
#Target stand basal area (m^2^)#	0

Group selection parameters	
#Management Area ID#	5 (e.g.)
#Ranking algorithm#	6
#Entry year#	5
#Reentry year#	5
#Minimum stand harvest basal area (m^2^)#	5
#remove largest tree first#	3
#Proportion of management area to harvested#	0.15
#proportion of stand to be harvested#	0.2
#Mean group size#	1
#Standard deviation of group size#	1

## Results

### Landscape initialization and model calibration using FIA data

Our results indicated that the initialized forest conditions constructed from FIA data for 1978 captured the historical species composition of oak-dominated forests at 1978 reasonably well. There were no significant differences in species density (χ2 = 1.93, df = 5,P = 0.86) and basal area (χ2 = 1.40, df = 5,P = 0.92) at the landscape scale nor by landtype (southwest: χ2 = 2.55 df = 5,P = 0.77; χ2 = 1.48, df = 5,P = 0.92; northeast χ2 = 2.82, df = 5,P = 0.73; χ2 = 1.18, df = 5,P = 0.95).

Prior to calibration, the predicted species density and basal area projected from 1978 to 2008 differed significantly from observed values reported from 2008 FIA data. Therefore, we made iterative adjustments to a model parameter, number of potential germinating seeds for each species to ensure predicted density and basal area from 1978 to 2008 matched observed values from FIA data. Following this calibration, there were no significant differences between LANDIS PRO predicted and observed 2008 values in species density (landscape: *χ^2^* = 1.85, *df* = 5,P = 0.87*;* southwest landtypes: *χ^2^* = 1.04, *df* = 5,P = 0.96*;* northeast landtypes: *χ^2^* = 2.68, *df* = 5,P = 0.75), nor in basal area (landscape: *χ^2^* = 2.61, *df* = 5,P = 0.76*;* southwest landtypes: *χ^2^* = 3.70, *df* = 5,P = 0.59; northeast landtypes: *χ^2^* = 1.85, *df* = 5,P = 0.87). Thus, the calibrated model parameters predicted reasonable outcomes.

### Effects of harvest alternatives on forest composition

**Figure 2:**
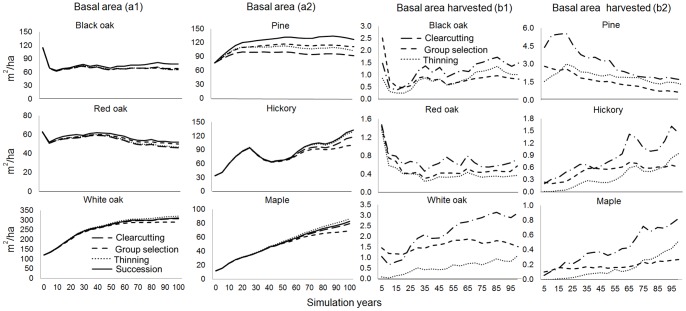
Simulated basal area (a1, a2) and harvested basal area by species (b1, b2) for 100 years simulation under the four harvest alternatives (one no-harvest alternative with only natural succession).

At 2008, white oaks were dominant and accounted for 30 percent of total basal area across the landscape (Figure 2-a1, a2), whereas red oaks comprised 40 percent of total basal area. Hickories and maples were common across the landscape and comprised 10 percent and 5 percent of total basal area, respectively. Pines, which tended to be spatially clustered, comprised 15 percent of total basal area. Under the four harvest alternatives, there were gradual increases in basal area of white oaks, pines, hickories, and maples (Figure 2- a1, a2). Our model predictions indicated that white oaks would continue to dominate the landscape for the next 100 years. In contrast, red oaks had a slight decrease in basal area after 2080, because a large proportion of red oaks that became established in the early to mid1900’s reached maximum longevity, died, and were replaced by young trees. The predicted basal area of maples that were shade-tolerant gradually increased over the next 100 years. These predicted successional trajectories were consistent with previous studies in oak forests in central hardwood forest regions: oak-dominated forests gradually shifted towards a greater proportion of longer-lived white oak, and shade-tolerant species such as sugar maple [16].

Compared to no-harvest alternative, the species basal area was reduced under the harvest alternatives, with the exceptions that basal area of white oaks and maples was slightly higher under the thinning alternative than that under the no-harvest alternative. These exceptions were mainly due to the competitiveness of white oaks and maples. Relatively shade -tolerant species such as white oak and sugar maple can become established once the growing space was released by thinning, which at the mean time prevented other shade-intolerant species from becoming established.

**Figure 3:**
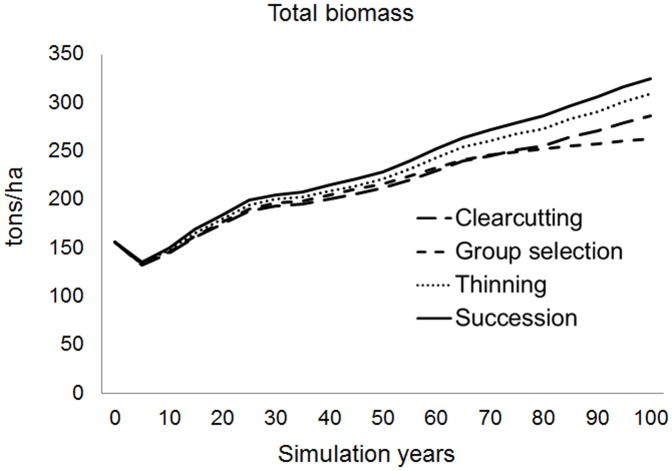
Predicted biomass accumulation of all species for 100 years simulation under the four harvest alternatives (one no-harvest alternative with only natural succession).

The amounts of basal area harvested for each species group were also tracked. There were more amounts of basal area harvested under the group selection and clearcutting alternatives (Figure 2 - b1 b2). The trends of species basal area harvested were reflected in the residual basal area by species for live trees. Proportionate increases or decreases in harvested basal area followed the relative abundance of species across the landscape. Thus, for a given species group, the basal area harvested tended to increase over time as the overall basal area of that species increased across the landscape. Basal area and biomass reductions were greatest in the clearcutting alternative, followed by the group selection and thinning alternatives. The thinning alternative accumulated the most living biomass over time, followed by the group selection and clearcutting alternatives (Figure 3). Greater cumulative forest harvesting thus reduced the quantity of living biomass across the landscape.

### The spatial distribution of potential oak decline high risk sites

**Figure 4:**
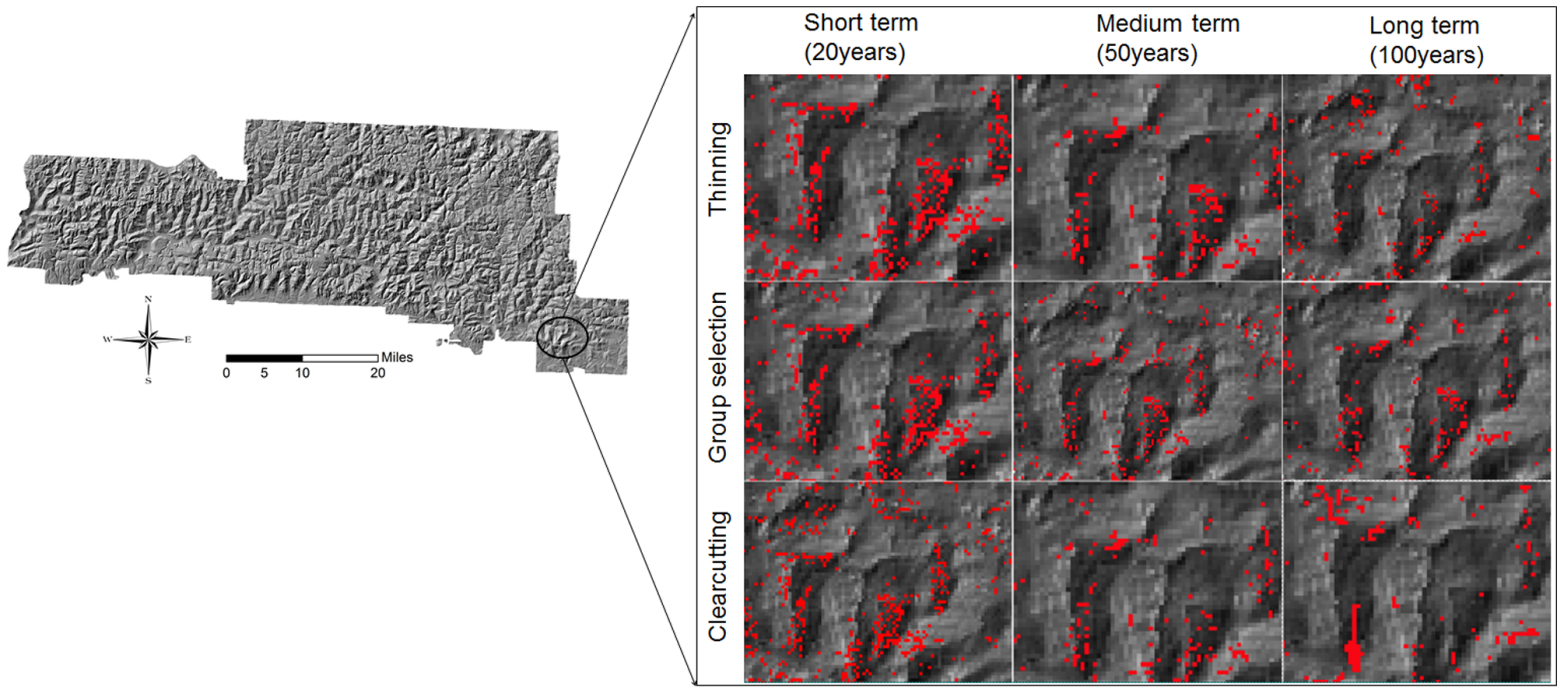
The magnified proportion of simulated landscape to demonstrate the spatial distribution of high risk sites for oak decline under the three harvest alternatives.

The potential oak decline high risk sites were spatially delineated for the whole landscape over time. The spatial delineations showed that these high risk sites decreased significantly over time under the three harvest alternatives (Figure 4). At the landscape scale, the spatial patterns of high risk sites were scattered across the landscape and similar among the three harvest alternatives. The scattered patterns were associated with the scattered distribution of the red oaks and site quality that in turn were determined by dissected topography. However, at the finer scale, there were smaller harvest patches under the thinning alternative compared to the clearcutting and group selection alternatives (Figure 4).

### Effects of harvest alternatives on mitigating the potential oak decline risk

**Figure 5:**
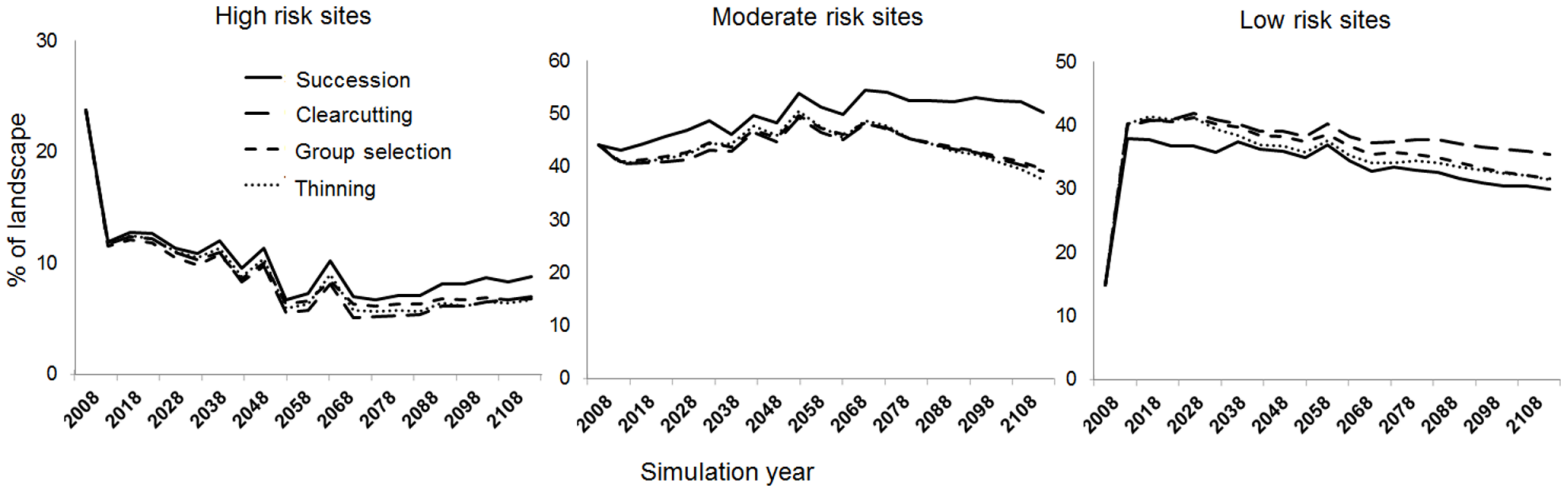
The potential high-, moderate-, and low-risk for oak decline under the four harvest alternatives (one no-harvest alternative with only natural succession).

Over the 100-years simulations, the potential oak decline high risk sites would decrease strongly under the four harvest alternatives (Figure 5). But the trends were reversed for the remaining proportion of sites in low risk. At simulation year 0 (2008), about 25% of the landscape was ranked as high risk. However, in the first iteration of the simulation, there was an abrupt drop in high risk sites and a corresponding increase of low risk sites. This occurred as the model equilibrated for initial conditions. In this case, the self-thinning algorithms removed trees growing under conditions of severe competition, which in turn affected all treatments equally. The rate of self-thinning in one of the iterations was faster than what actually would have occurred in the field. However, the long-term trends quickly stabilized and they were appropriate to compare the relative differences among simulated alternatives.

**Figure 6:**
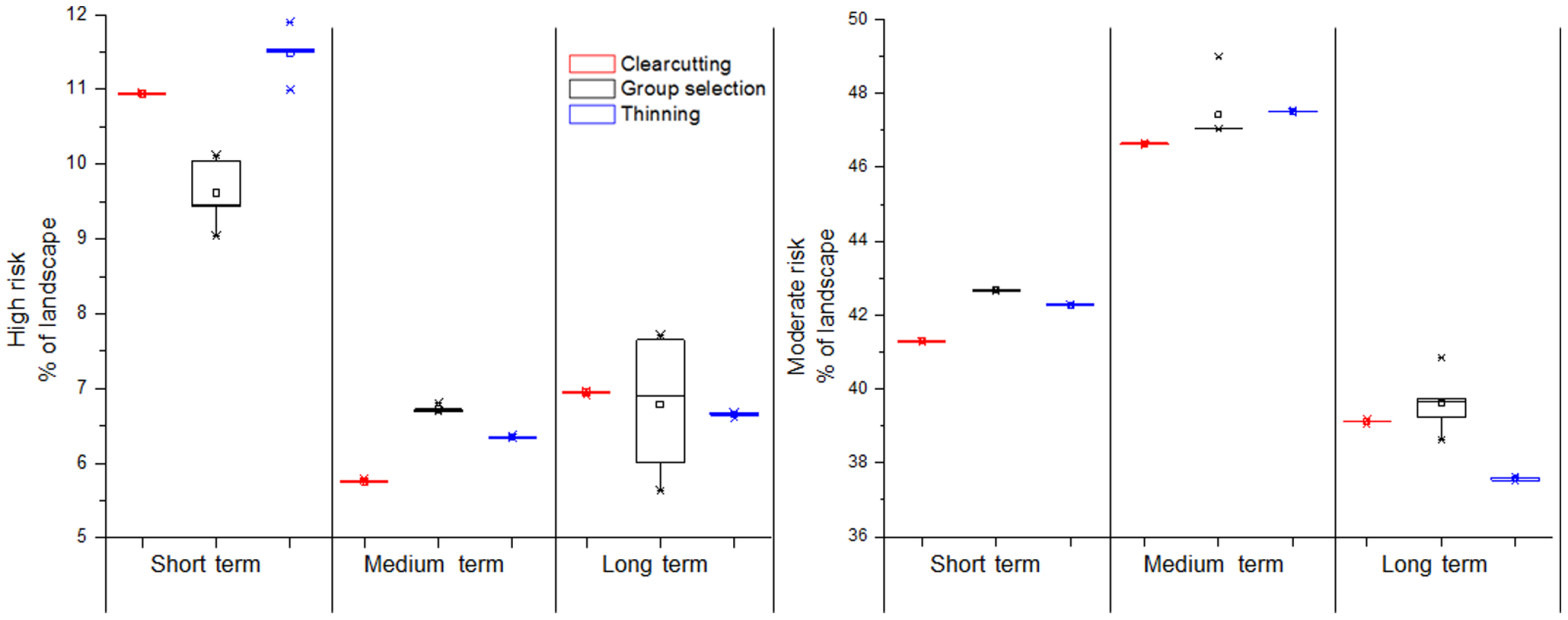
The potential high- and moderate-risk areas for oak decline under the three harvest alternatives in the short, medium, and long terms.

Our results showed that forest harvesting played a role in reducing the proportion of sites in high or moderate risk compared to the no-harvest alternative (Figure 5). The differences between the harvest alternatives and the no-harvest alternative in moderate- and high-risk sites can be as large as 13% and 3% in the long term, respectively. However, the effectiveness varied among the three harvest alternatives in short, medium, and long terms (Figure 6). In the short term, the group selection alternative was the most effective option for reducing the proportion of high risk sites followed by the clearcutting and then thinning alternatives (9.56%, 10.95%, and 11.51% of landscape, respectively). After 50-years simulation, the proportion of high risk sites decreased more in the clearcutting alternative than in the thinning and group selection alternatives (5.62%, 6.85%, and 6.46% of landscape, respectively). After 100 years of simulation, the predicted proportion of high risk sites became less discernible for the three harvest alternatives. For reducing the proportion of moderate risk sites, the clearcutting alternative was the more effective option compared to the thinning and group selection alternatives (e.g., 41.16%, 42.72%, 42.11% of landscape in the short term, respectively).

## Discussion

Results showed that the potential oak decline sites decrease strongly in the next five decades regardless irrespective of harvest alternatives. This result reveals that the oak decline is a natural process, which has a natural cycle of development for predisposed oak stands [16], [39]. Predisposing factors including tree age, species composition, and stand density will change as a result of tree growth, seedling establishment, and mortality. At the beginning of the simulation (2008), oak forests in this study area range from 70 to 100 years of age with extremely high stand density [25]. In the next five decades, intense self-thinning arising from strong competition will result in high tree mortality. Thus, the natural succession from this stage will eventually lead to the decrease in oak decline. Understanding this trend is significant for forest managers and planners to develop silvicultural prescriptions and long-term management plans in the context of natural succession.

Our results show that forest harvesting does play a role in managing oak decline. Harvest reduced the proportion of high risk sites by about 3% and moderate risk sites by about 13%, which corresponded to about 1,300 ha and 5,600 ha in this study area, respectively. These amounts of forest are significant since the proportion of most National Forests in U.S. can be treated is about 1–5% per decade [38]. In addition, the effectiveness varied among the three harvest alternatives. In general, the group selection and clearcutting alternatives were most effective in mitigating oak decline in the short and medium terms, respectively. However, differences among the effects of the three harvest alternatives became less significant in the long term. The group selection alternative is effective in removing red oaks from scattered sites caused by highly dissected terrains and the scattered distributed groups of red oaks.

Harvest parameters in the three harvest alternatives including species priority, harvest rotation, harvest area, and residual stand basal area may also affect how harvest alternatives mitigate oak decline. The harvest alternatives in our study were derived from the current management plan of Ozark-St. Francis National Forest. This plan included the balanced strategies of managing both oak regeneration and oak decline [38]. According to this plan, black and red oak were ranked third and fourth in the thinning alternative following hickory and maple, because removing competitive species such as maple is needed for promoting oak regeneration. If black and red oak are given the highest priority for harvesting, the oak decline risk would be expected to further reduce under the thinning alternative. However, in the long run, oak decline risk sites may greatly decrease not only due to the prioritization but also due to the lack of oak regeneration.

We show that we can apply a spatially explicit forest landscape modeling approach to investigate the effects of management alternatives at the landscape scale. The recent advances in FLMs such as LANDIS PRO, which outputs species density and basal area, make it possible to derive response variables directly related to oak decline (e.g. oak decline risk sites). With the advent of new measurement techniques and nearly three decades of additional inventory data accumulation, FIA data that include large-scale spatiotemporal data of forest composition and structure provide tremendous potential for FLMs to use these data to initialize forest landscapes and calibrate model parameters [26]. In our study, FIA data were combined with LANDIS PRO FLM to initialize forest landscape and calibrate model parameter before predicting future changes towards an era of model-data infusion, an emerging area of research in ecology [40]–[41].With such a landscape modeling approach, we were able to spatially delineate oak decline sites in this study. Results indicated that the high risk oak decline sites were spatially scattered across the landscape, which was largely associate with highly dissected topography and high basal areas of the decline-susceptible red oaks. The scattered patterns pose challenges to traditional silvicultural treatments [20]. For example, managements that focus on removing susceptible species and age cohorts may be difficult, because the aggregated large patches of vulnerable sites are often not available across the landscape. However, because relatively large “hot spots” of high risk can be spatially identified, risk managements could be effectively focused there.
